# Frailty indices predict mortality, complications and functional improvements in supratentorial meningioma patients over 80 years of age

**DOI:** 10.1007/s11060-024-04780-6

**Published:** 2024-09-04

**Authors:** Christoph Schwartz, Moritz F. Ueberschaer, Ilari Rautalin, Jürgen Grauvogel, Marco Bissolo, Waseem Masalha, Christine Steiert, Oliver Schnell, Jürgen Beck, Florian Ebel, David Bervini, Andreas Raabe, Thomas Eibl, Hans-Herbert Steiner, Karl-Michael Schebesch, Nathan A. Shlobin, Khizar R. Nandoliya, Mark W. Youngblood, James P. Chandler, Stephen T. Magill, Alexander Romagna, Jens Lehmberg, Manuel Fuetsch, Julian Spears, Arwin Rezai, Barbara Ladisich, Matthias Demetz, Christoph J. Griessenauer, Mika Niemelä, Miikka Korja

**Affiliations:** 1 Department of Neurosurgery, Helsinki University and Helsinki University Hospital, Helsinki, Finland; 2 Department of Neurosurgery, University Hospital Salzburg, Paracelsus Medical University, Salzburg, Austria; 3 Present Address: The National Institute for Stroke and Applied Neurosciences, Auckland University of Technology, Auckland, New Zealand; 4 Department of Neurosurgery, Medical Center, University of Freiburg, Freiburg, Germany; 5 Present Address: Department of Neurosurgery, University Hospital Erlangen, University of Erlangen–Nuremberg, Erlangen, Germany; 6 Department of Neurosurgery, Inselspital, Bern University-Hospital, Bern, Switzerland; 7 Present Address: Department of Neurosurgery, University of Basel, Basel, Switzerland; 8 Department of Neurosurgery, Paracelsus Medical University, Klinikum Nuremberg, Nuremberg, Germany; 9 Department of Neurological Surgery, Northwestern University, Chicago, IL 60601 USA; 10 Feinberg School of Medicine, Northwestern University, 676 North St Clair Street, Suite 2210, Chicago, IL 60601 USA; 11 Department of Neurosurgery, München Klinik Bogenhausen, Munich, Germany; 12 Division of Neurosurgery, St. Michael’s Hospital, University of Toronto, Toronto, Canada; 13 Present Address: Department of Spine and Scoliosis Surgery, Artemed Surgical Clinic Munich South, Munich, Germany; 14 Present Address: Department of Neurosurgery, University Hospital St. Pölten, St. Pölten, Austria; 15 Present Address: Department of Neurosurgery, Medical University of Innsbruck, Innsbruck, Austria; 16 Present Address: Department of Neurosurgery, University Hospital Salzburg, Paracelsus Medical University Salzburg, Ignaz-Harrer-Str. 79, 5020 , Salzburg, Austria; 17 Present Address: Department of Neurosurgery, München Klinik Bogenhausen, Munich, Germany

**Keywords:** Elderly, Functional outcome, Meningioma, Modified 5 (mFI-5) and 11 (mFI-11) Factor Frailty Indices, Mortality

## Abstract

**Purpose:**

To assess whether the Modified 5 (mFI-5) and 11 (mFI-11) Factor Frailty Indices associate with postoperative mortality, complications, and functional benefit in supratentorial meningioma patients aged over 80 years.

**Methods:**

Baseline characteristics were collected from eight centers. Based on the patients’ preoperative status and comorbidities, frailty was assessed by the mFI-5 and mFI-11. The collected scores were categorized as “robust (mFI=0)”, “pre-frail (mFI=1)”, “frail (mFI=2)”, and “significantly frail (mFI≥3)”. Outcome was assessed by the Karnofsky Performance Scale (KPS); functional benefit was defined as improved KPS score. Additionally, we evaluated the patients’ functional independence (KPS≥70) after surgery.

**Results:**

The study population consisted of 262 patients (median age 83 years) with a median preoperative KPS of 70 (range 20 to 100). The 90-day and 1-year mortality were 9.0% and 13.2%; we recorded surgery-associated complications in 111 (42.4%) patients. At last follow-up within the postoperative first year, 101 (38.5%) patients showed an improved KPS, and 183 (69.8%) either gained or maintained functional independence. “Severely frail” patients were at an increased risk of death at 90 days (OR 16.3 (CI95% 1.7-158.7)) and one year (OR 11.7 (CI95% 1.9-71.7)); nine (42.9%) of severely frail patients died within the first year after surgery. The “severely frail” cohort had increased odds of suffering from surgery-associated complications (OR 3.9 (CI 95%) 1.3-11.3)), but also had a high chance for postoperative functional improvements by KPS≥20 (OR 6.6 (CI95% 1.2-36.2)).

**Conclusion:**

The mFI-5 and mFI-11 associate with postoperative mortality, complications, and functional benefit. Even though “severely frail” patients had the highest risk morbidity and mortality, they had the highest chance for functional improvement.

**Supplementary information:**

The online version contains supplementary material available at 10.1007/s11060-024-04780-6.

## Introduction

The number of elderly and very old patients with symptomatic supratentorial meningiomas requiring neurosurgical evaluation and treatment is likely to increase [1–4]. Older age at surgery and associated frailty are significant predictors for poor outcomes in cranial neurosurgery; hence, optimal decision-making for these patients remains a difficult undertaking [5–19]. Patient frailty has recently become the focus of surgical treatment guidance, and several scores have been proposed to facilitate preoperative risk assessment [19–34]. While most frailty classifications such as the Canadian Study of Health and Aging Modified Frailty Index are either not suitable for neurosurgical patients or simply too extensive for easy implementation in outpatient counseling, there are also more compact scores such as the Modified 5 (mFI-5) and 11 (mFI-11) Factor Frailty Indices that can be determined concisely during a single outpatient visit based on the patients’ comorbidities and functional dependency (Table 1) [19–34]. However, the amount of previous evidence on such scores especially among very old meningioma patients has remained limited.

**Table 1:** Frailty indices and recorded comorbidities

*Modified 5 Factor Frailty Index (mFI-5)*	*Modified 11 Factor Frailty Index (mFI-11)*
Functional dependent	Cognitive impairment
History of diabetes	Delirium
Chronic obstructive pulmonary disease (COPD)	Myocardial infarction
Congestive heart failure (CHF)	History of diabetes
Hypertension	Congestive heart failure (CHF)
-	History of stroke
-	Hypertension
-	Cerebrovascular problems
-	Chronic obstructive pulmonary disease (COPD)
-	Coronary artery disease
-	Functional dependent
*Sum score mFI-5*	*Sum score mFI-11*
0 = robust	0 = robust
1 = pre-frail	1 = pre-frail
2 = frail	2 = frail
≥3 = severely frail	≥3 = severely frail
Most common comorbidities in the study population*	
Hypertension (n):	179 (53.9%)
Coronary heart disease (n):	49 (14.8%)
Diabetes (n):	42 (12.7%)
COPD (n):	18 (5.4%)
Stroke/cerebrovascular problems (n):	14 (4.2%)
Myocardial infarction (n):	12 (3.6%)

*CHF* Congestive heart failure; *COPD* Chronic obstructive pulmonary disease; *KPS* Karnofsky Performance Scale; *PTBE* Peritumoral brain edema; *Percentages calculated as proportion of all recorded relevant co-morbidities (n=332) for the entire study population; multiple entries possible per patient

We previously introduced an easy-to-use decision-support tool for supratentorial meningioma patients aged 80 years or older to identify patients who would most likely benefit from tumor resection concerning their neurofunctional outcomes [35, 36]. Our three-tiered decision-support tool is based on the patients’ preoperative functional status and the extent of peritumoral brain edema (PTBE) volume [35, 36]. Prior analyses indicated that especially patients with poor preoperative functional status and large PTBE should be considered for surgery, since they displayed a significant chance for functional improvement without extensive surgery-associated complications [35, 36]. In this context, we performed a follow-up study to evaluate whether the mFI-5 and mFI-11 indices may also be useful alternatives to our classification system in predicting patients’ improvements after meningioma resection. Furthermore, the potential prediction of postoperative mortality, surgery-associated complications, and occurrence of new neurological deficits as well as the patients’ capability to live at home were assessed.

## Methods

### Data collection and frailty indices assessment

Overall, 262 supratentorial meningioma patients who underwent neurosurgical operation at the aged 80 years or older were identified within the existing framework of our multicenter database [36]. In brief, we collected demographic (i.e. age, sex, comorbidities etc.) and radiological data (i.e. tumor location, tumor and PTBE volumes etc.) of patients, who had undergone first-ever tumor resection at six European and two North American high-volume neurosurgical centers [36]. The patients’ functional status was evaluated pre- and postoperatively by the Karnofsky Performance Scale (KPS) [37]. In accordance with the prior study, tumor and PTBE volumes were graded as large (>50 cm^3^), medium (10-50 cm^3^), and small (<10 cm^3^) as assessed by three-dimensional volumetric measurements of contrast-enhanced T1-weighted and T2-weighted magnetic resonance imaging using neuronavigation software available at the participating centers [35, 36]. Based on the patients’ comorbidities recorded in the electronic health records, we calculated the sum scores for the mFI-5 and mFI-11 indices (Table 1). Following prior recommendations, the sum mFI scores were categorized for further analyses as follows: robust (mFI=0), pre-frail (mFI=1), frail (mFI=2), and severely frail (mFI≥3) [22, 34].

The study was conducted in accordance with the Declaration of Helsinki, and the de-identified data were shared with the central site. The study was approved by the institutional review board.

### Outcome measurements

This study’s main outcome parameters were the patients’ functional benefit from surgery, mortality within the first year after surgery, and the risk for surgery-associated complications. The decision to focus on the surgical results within the first postoperative year was selected to minimize the potential confounding of age-related diseases on functional outcome assessment. In addition, our previous analyses have shown that surgery-related mortality occurs mostly within this timeframe [7, 35, 36]. For outcome assessment, the preoperative KPS was compared to the last recorded KPS within the first postoperative year for each patient. The resulting differences between the KPS values were then categorized as improved versus unchanged and worse; separate analyses were made for any (i.e. KPS increase of ≥10) and major (i.e. KPS increase of ≥20) improvements. Additionally, we assessed outcome with regard to the patients’ functional independence (i.e. KPS≥70); postoperative outcome was stratified as functional independence gained (preoperative KPS<70 and postoperative KPS≥70), maintained (pre- and postoperative KPS≥70), neither (pre- and postoperative KPS<70), and lost (preoperative KPS≥70 but postoperative KPS<70).

In addition to one-year mortality, we recorded 90-day mortality as well as any surgery-associated complications and new neurological deficits. Surgery-associated complications included intracerebral hemorrhage, all postoperative local or systemic infections requiring treatment, pulmonary embolism, cardiovascular/respiratory failure, any revision surgery, occurrence of new neurologic deficits, and postoperative epilepsy. Another outcome assessment was the patients’ postoperative capability to live at home, i.e. whether patients were living/returned to their homes after tumor resection or had to be accommodated in a care center.

### Statistics

Descriptive statistics including median (range) and frequencies were used to characterize the patient population and subgroups. Association analyses were conducted depending on the data format (i.e. chi-squared and Kruskall-Wallis), and binary logistic regression analyses to calculate odds ratios (ORs) with 95% confidence interval (CI) for outcome measurements. The regression analyses were adjusted for patients’ age, sex, tumor and PTBE volume categories as these parameters had been previously identified to associate with the patients’ preoperative status and functional outcome/postoperative mortality [7, 35, 36]. Missing data/values were excluded from the corresponding statistical analyses. A p-value of <0.05 indicated statistical significance. All statistical analyses were performed using IBM SPSS Statistics version 29.

## Results

### Patients and tumors

Our cohort included 262 patients (57.3% females), who underwent surgery between 2009 and 2022. The median age at surgery was 83 years (range 80 to 96), the median preoperative KPS was 70 (range 20 to 100) and 168 (50.6%) patients were functionally independent (Table 2). In total, we recorded 332 specific comorbidities affecting 229 (87.4%) patients in the entire study population, and 91 (34.7%) patients suffered from multiple comorbidities as assessed by the mFI-5 and mFI-11 indices (Table 1). The most common comorbidities were hypertension (n=179), coronary heart disease (n=49), and diabetes (n=42) (Table 1).

**Table 2:** Patient and tumor characteristics

*Patient characteristics*									
	All patients (n=262)	Modified 5 Factor Frailty Index (mFI-5)				Modified 11 Factor Frailty Index (mFI-11)			
		*Robust “mFI=0” (n=44)*	*Pre-frail “mFI=1” (n=121)*	*Frail “mFI=2”* *(n=71)*	*Severely frail “mFI≥3” (n=26)*	*Robust “mFI=0” (n=36)*	*Pre-frail “mFI=1” (n=104)*	*Frail “mFI=2”* *(n=65)*	*Severely frail “mFI≥3” (n=57)*
Median age (range) in years:	83.0 (80.0-96.0)	83.0 (80.0-88.0)	83.0 (80.0-95.0)	83.0 (80.0-96.0)	83.0 (80.0-93.0)	83.0 (80.0-88.0)	83.0 (80.0-95.0)	83.0 (80.0-90.0)	83.0 (80.0-96.0)
Females:	150 (57.3%)	20 (45.5%)	73 (60.3%)	43 (60.6%)	14 (53.8%)	17 (47.2%)	64 (61.5%)	39 (60.0%)	30 (52.6%)
Median preoperative KPS (range):	70 (20-100)	80 (60-100)	80 (40-100)	60 (20-100)	40 (30-80)	80 (70-100)	80 (40-100)	70 (20-100)	50 (30-100)
Functional independence (n):	168 (50.6%)	43 (97.7%)	99 (81.8%)	24 (33.8%)	2 (7.7%)	36 (100.0%)	83 (79.8%)	33 (50.8%)	16 (28.1%)
*Tumor characteristics*									
* Most common locations*									
Convexity (n):	93 (28.0%)	20 (45.5%)	41 (33.9%)	23 (32.4%)	9 (34.6%)	16 (44.4%)	37 (35.6%)	20 (30.8%)	20 (35.1%)
Sphenoid wing (n):	61 (18.4%)	7 (15.9%)	25 (20.7%)	21 (29.6%)	8 (30.8%)	6 (16.7%)	22 (21.2%)	18 (27.7%)	15 (26.3%)
Falx (n):	37 (11.1%)	9 (20.5%)	17 (14.0%)	8 (11.3%)	3 (11.5%)	8 (22.2%)	14 (13.5%)	11 (16.9%)	4 (7.0%)
Anterior fossa/ skullbase (n):	37 (11.1%)	7 (15.9%)	20 (16.5%)	9 (12.7%)	1 (3.8%)	6 (16.7%)	15 (14.4%)	7 (10.8%)	9 (15.8%)
Middle fossa/ skullbase (n):	27 (8.1%)	1 (2.3%)	15 (12.4%)	9 (12.7%)	2 (7.7%)	0 (0.0%)	14 (13.5%)	7 (10.8%)	6 (10.5%)
Left lateralization (n):	130 (51.0%)	19 (44.2%)	62 (53.4%)	38 (54.3%)	11 (42.3%)	16 (45.7%)	52 (51.0%)	36 (58.1%)	26 (46.4%)
* Measurements and volumes*									
Maximum tumor diameter ≥5cm (n):	99 (35.5%)	18 (41.9%)	41 (34.7%)	33 (47.1%)	7 (26.9%)	15 (42.9%)	41 (40.2%)	20 (31.7%)	23 (40.4%)
Median tumor volume (range) in cm^3^:	30.2 (0.5-215.0)	33.8 (2.2-149.2)	29.6 (0.7-142.6)	34.5 (0.5-215.0)	30.3 (1.3-127.5)	35.5 (2.2-149.2)	30.2 (0.7-142.6)	26.6 (1.8-116.6)	32.9 (0.5-215.0)
Median PTBE volume (range) in cm^3^:	27.3 (0.0-408.9)	20.1 (0.0-197.1)	29.7 (0.0-408.9)	27.8 (0.0-275.3)	30.1 (0.0-188.5)	20.1 (0.0-143.4)	30.7 (0.0-408.9)	28.6 (0.0-275.3)	25.9 (0.0-196.5)
* Tumor volume categories*									
Small (<10 cm^3^) (n):	35 (13.5%)	3 (6.8%)	21 (17.6%)	9 (12.9%)	2 (7.7%)	2 (5.6%)	14 (13.6%)	10 (15.9%)	9 (15.8%)
Medium (10- 50 cm^3^) (n):	147 (56.8%)	28 (63.6%)	63 (52.9%)	37 (52.9%)	19 (73.1%)	22 (61.1%)	56 (54.4%)	39 (61.9%)	30 (52.6%)
Large (>50 cm^3^) (n):	77 (29.7%)	13 (29.5%)	35 (29.4%)	24 (34.3%)	5 (19.2%)	12 (33.3%)	33 (32.0%)	14 (22.2%)	18 (31.6%)
* PTBE volume categories*									
Small (<10 cm^3^) (n):	83 (32.0%)	18 (40.9%)	38 (31.9%)	20 (28.6%)	7 (26.9%)	15 (41.7%)	30 (29.1%)	20 (31.7%)	18 (31.6%)
Medium (10- 50 cm^3^) (n):	94 (36.3%)	12 (27.3%)	41 (34.5%)	29 (41.4%)	12 (46.2%)	10 (27.8%)	38 (36.9%)	24 (38.1%)	22 (28.6%)
Large (>50 cm^3^) (n):	82 (31.7%)	14 (31.8%)	40 (33.6%)	21 (30.0%)	7 (26.9%)	11 (30.6%)	35 (34.0%)	19 (30.2%)	17 (29.8%)

^*^Data not available for all patient subgroups; percentages calculated for available valid data

The most frequent symptoms triggering initial cerebral imaging/meningioma diagnosis were motor deficits (n=52), headache/vertigo (n=48), and visual deficits (n=46). In the majority of patients (92.4%), tumor resection was deemed indicated due to neurological deficits and/or significant mass effect.

The median tumor and PTBE volumes were 30.2 cm^3^ (range 0.5-215.0) and 27.3 cm^3^ (range 0.0-408.9). Furthermore, 55 (21.0%) tumors had no PTBE, and 99 (35.5%) tumors were giants (i.e. had a maximum diameter of ≥5cm in any plane) (Table 2). The most common tumor locations were the convexity (n=93), the sphenoid wing (n=61) as well as the falx (n=37); additionally, 64 tumors were classified as anterior and middle fossa skullbase meningiomas (Table 2). Postoperative MRI was available for assessment in 204 cases, and showed a residual tumor in 41 (20.1%) of those patients. The majority of tumors (69.1%) were graded as WHO grade 1 and only a small proportion (n=6) of tumors showed malignant histology (i.e. WHO grade 3); thus, a rather significant share of tumors (29.0%) showed grade 2 histopathology. Based on available follow-up data, thirteen patients underwent adjuvant radiotherapy and one patient received adjuvant radionuclide therapy.

### Frailty indices scores

According to the mFI-5 and the mFI-11 indices, median sum scores were 1 (range 0 to 4) and 1 (range 0 to 7), respectively. The majority of patients, 121 (46.2%) and 104 (29.7%) cases, respectively, were categorized as pre-frail by the mFI-5 and mFI-11 (Table 2). Significant differences between the frailty subgroups, as generated by the mFI-5 and mFI-11, were found with regard to the preoperative KPS and functional independence (all p<0.001, chi-squared); poor neurofunctional status associated with increased frailty sum scores in both indices (Table 2). No differences between the mFI-5 and mFI-11 cohorts regarding age at surgery (p=0.821 and p=0.542, Kruskal-Wallis), sex (p=0.332 and p=0.398, chi-squared), tumor location (p=0.105 and p=0.504, chi-squared), lateralization (p=0.095 and p=0.579, chi-squared), tumor volume (p=0.305 and p=0.615, chi-squared), and PTBE (p=0.643 and p=0.870, chi-squared) volume categories were recorded (Table 2).

### Functional outcome

Outcome assessment at last available follow-up within the first postoperative year were the following points in time; at discharge (n = 52), 3-6 months postoperatively (n = 90), and 12 months postoperatively (n = 120). At last follow-up within the first postoperative year, 101 of 262 (38.5%) patients showed any functional benefit from tumor resection by improvement in their KPS status (Table 3). Furthermore, major improvements were recorded for 52 (15.7%) patients, and functional independence was either gained or maintained by 183 (69.8%) patients (Table 3).

**Table 3:** Functional outcome, mortality, and complications

	All patients (n=262)	Modified 5 Factor Frailty Index (mFI-5)				Modified 11 Factor Frailty Index (mFI-11)			
		*Robust “mFI=0” (n=44)*	*Pre-frail “mFI=1” (n=121)*	*Frail “mFI=2”* *(n=71)*	*Severely frail “mFI≥3” (n=26)*	*Robust “mFI=0” (n=36)*	*Pre-frail “mFI=1” (n=104)*	*Frail “mFI=2”* *(n=65)*	*Severely frail “mFI≥3” (n=57)*
*Functional benefit at last follow-up within the first postoperative year*									
Any functional improvement (KPS≥10) (n):	101 (38.5%)	13 (29.5%)	45 (37.2%)	34 (47.9%)	9 (34.6%)	9 (25.0%)	42 (40.4%)	26 (40.0%)	24 (42.1%)
Major improvement (KPS≥20) (n):	52 (15.7%)	2 (4.5%)	21 (17.4%)	23 (32.4%)	6 (23.1%)	2 (5.6%)	17 (16.3%)	15 (23.1%)	18 (31.6%)
Unchanged (n):	87 (33.2%)	23 (52.3%)	42 (34.7%)	18 (25.4%)	4 (15.4%)	20 (55.6%)	36 (34.6%)	18 (27.7%)	13 (22.8%)
Worse (n):	74 (28.2%)	8 (18.2%)	34 (28.1%)	19 (26.8%)	13 (50.0%)	7 (19.4%)	26 (25.05)	21 (32.3%)	20 (35.1%)
*Functional independence (KPS≥70) at last follow-up within the first postoperative year*									
Gained (n):	45 (17.2%)	1 (2.3%)	15 (12.4%)	23 (32.4%)	6 (23.1%)	0 (0.0%)	15 (14.4%)	15 (23.1%)	15 (26.3%)
Maintained (n):	138 (52.7%)	35 (79.5%)	83 (68.6%)	16 (22.5%)	4 (15.4%)	29 (80.6%)	72 (69.2%)	24 (36.9%)	13 (22.8%)
Neither (n):	43 (16.4%)	0 (0.0%)	7 (5.8%)	23 (32.4%)	13 (50.0%)	0 (0.0%)	5 (4.8%)	17 (26.2%)	21 (36.8%)
Lost (n):	36 (13.7%)	8 (18.2%)	16 (13.2%)	9 (12.7%)	3 (11.5%)	7 (19.4%)	12 (11.5%)	9 (13.8%)	8 (14.0%)
Postoperative mortality, surgery-associated complications and postoperative new deficits*									
Within 90- days (n):	20 (9.0%)	1 (2.5%)	5 (5.2%)	7 (11.1%)	7 (30.4%)	1 (2.9%)	5 (6.1%)	3 (5.2%)	11 (22.4%)
Within 12- months (n):	25 (13.2%)	2 (5.9%)	5 (6.0%)	9 (17.3%)	9 (42.9%)	2 (9.6%)	6 (8.5%)	4 (8.3%)	13 (31.0%)
Complications (n):	111 (42.4%)	10 (22.7%)	50 (41.3%)	37 (52.1%)	14 (53.8%)	9 (25.0%)	42 (40.4%)	29 (44.6%)	31 (54.4%)
New deficits (n):	46 (17.6%)	4 (9.1%)	24 (19.8%)	12 (16.9%)	6 (23.1%)	4 (11.1%)	20 (19.2%)	10 (15.4%)	12 (21.1%)
Patients’ ability to live at home after surgery*									
Living at home (n):	117 (73.1%)	22 (66.7%)	64 (80.0%)	24 (72.7%)	7 (50.0%)	19 (67.9%)	53 (81.5%)	23 (65.7%)	22 (68.8%)

^*^Data not available for all patient subgroups; percentages calculated for available valid data

The observed 90-day and 1-year mortality rates were 9.0% and 13.2% for the entire study population (Table 3). We recorded surgery-associated complications in 111 (42.4%) of patients. The most frequent complications were new neurological deficits, mostly affecting motor and language function, recorded for 25 (9.5%) patients, systemic infections and pneumonia in 14 (5.3%) patients, and intracerebral hemorrhage in eleven (4.2%) patients (Table 3). Data on the patients’ capability to live at home after surgery was available for 160 patients; of those 117 (73.1%) patients were able to live at home postoperatively, whereas 43 (26.9%) were dependent on care centers/hospitals (Table 3). The median postoperative KPS was 90 for patients living at home, and 70 for those requiring accommodation in a care center.

**Fig. 1:**
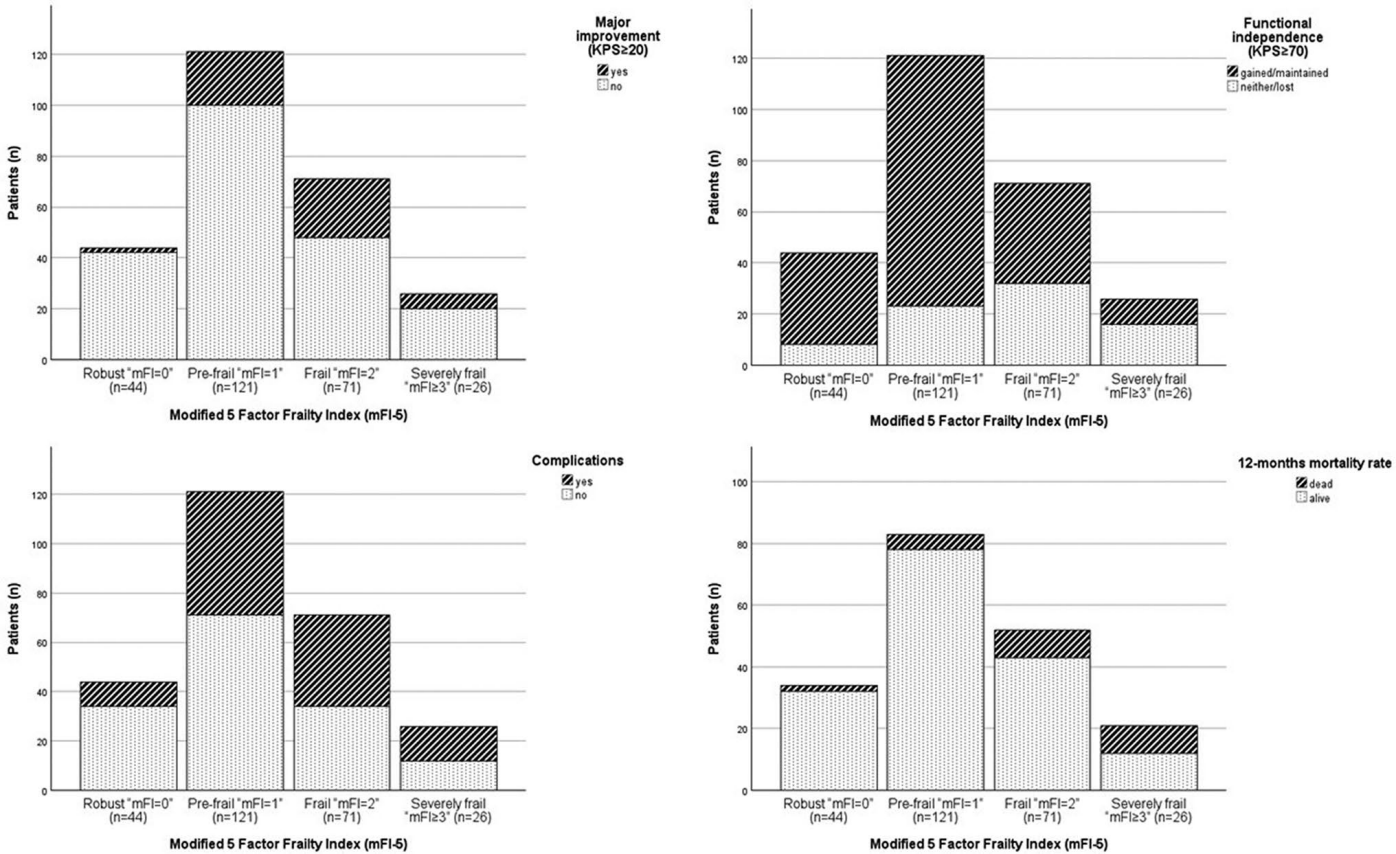
Patient frailty and outcome

The highest percentages of patients who showed major functional improvement and gained functional independence postoperatively were seen in the frail and severely frail subgroups (Fig. 1, Table 3). Furthermore, the highest rate of postoperative mortality and occurrence of surgery-associated complications were recorded for severely frail patients (Fig. 1, Table 3). All outcome parameters stratified by the mFI-5 and mFI-11 indices are demonstrated in Table 3.

Post hoc analyses of the subgroup of skullbase meningioma patients gave the following results; 25 (39.1%) patients showed postoperative improvement, and 44 (81.5%) patients maintained or gained functional independence within the first year after surgery (Supplementary Table 1). Additionally, 30 (46.9%) patients suffered from surgery-associated complications, and eleven (17.2%) patients had postoperative new deficits (Supplementary Table 1). Thus, the risk of both parameters was not significantly increased compared to patients with non-skullbase tumors (p=0.401 and p=0.929, chi-squared). Detailed outcome data of patients with skullbase meningiomas are shown in Supplementary Table 1.

### Statistics

Logistic regression (adjusted for age, sex, tumor and PTBE volumes) showed that the frail (OR 10.1 (CI95% 2.2-46.3)) and the severely frail patients (OR 6.6 (CI95% 1.2-36.2)) had significantly higher odds for major functional improvements compared to robust patients based on mFI-5 subgroups (Fig. 1, Tables 3 and 4). Similarly, severely frail patients had an increased risk of death (OR 16.3 (CI95% 1.7-158.7)) within 90 days and one year (OR 11.7 (CI95% 1.9-71.7)) after surgery (Fig. 1, Tables 3 and 4). Furthermore, the severely frail cohort had increased odds of suffering from surgery-associated complications (OR 3.9 (CI 95%) 1.3-11.3)) (Fig. 1, Tables 3 and 4). Similar associations were recorded for the mFI-11 subgroup analyses (Tables 3 and 4). Statistical analyses for the subgroup of patients with skullbase meningiomas are shown in Supplementary Table 2.

**Table 4:** Statistics

*Modified 5 Factor Frailty Index (mFI-5)*								
Parameter	Any functional Improvement (KPS≥10)	Major improvement (KPS≥20)	Gained/maintained functional independence (KPS≥70)	90-day mortality*	1-year mortality*	Surgery-associated complications	New neurological deficits	Capability to live at home*
Robust “mFI=0”:	[Reference] OR (CI95%)	[Reference] OR (CI95%)	[Reference] OR (CI95%)	[Reference] OR (CI95%)	[Reference] OR (CI95%)	[Reference] OR (CI95%)	[Reference] OR (CI95%)	[Reference] OR (CI95%)
Pre-frail “mFI=1”:	1.4 (0.7-3.0)	*4.5 (1.0-20.5)*	1.0 (0.4-2.4)	2.8 (0.3-26.6)	1.2 (0.2-7.6)	*2.5 (1.1-5.7)*	2.7 (0.9-8.4)	1.9 (0.7-5.2)
Frail “mFI=2”:	2.1 (0.9-4.7)	*10.1 (2.2-46.3)*	0.3 (0.1-0.7)	4.7 (0.5-42.5)	3.4 (0.6-18.3)	*3.7 (1.6-8.9)*	2.0 (0.6-6.7)	1.1 (0.3-3.3)
Severely frail “mFI≥3”:	1.3 (0.5-3.7)	*6.6 (1.2-36.2)*	0.1 (0.0-0.5)	*16.3 (1.7-158.7)*	*11.7 (1.9-71.7)*	*3.9 (1.3-11.3)*	2.7 (0.7-10.9)	0.5 (0.1-2.1)
*Modified 11 Factor Frailty Index (mFI-11)*								
Robust “mFI=0”:	[Reference] OR (CI95%)	[Reference] OR (CI95%)	[Reference] OR (CI95%)	[Reference] OR (CI95%)	[Reference] OR (CI95%)	[Reference] OR (CI95%)	[Reference] OR (CI95%)	[Reference] OR (CI95%)
Pre-frail “mFI=1”:	2.1 (0.9-4.9)	3.3 (0.7-15.1)	1.3 (0.5-3.5)	2.6 (0.3-24.4)	1.4 (0.2-8.2)	2.2 (0.9-5.2)	2.0 (0.6-6.5)	2.2 (0.8-6.5)
Frail “mFI=2”:	1.9 (0.8-4.9)	*5.6 (1.2-26.5)*	0.3 (0.1-0.9)	2.5 (0.2-26.7)	1.6 (0.2-10.0)	*2.7 (1.1-6.9)*	1.6 (0.5-5.7)	0.7 (0.2-2.2)
Severely frail “mFI≥3”:	2.3 (0.9-5.7)	*8.2 (1.7-38.5)*	0.2 (0.1-0.6)	*9.5 (1.1-84.8)*	*5.7 (1.1-31.0)*	*3.7 (1.5-9.6)*	2.1 (0.6-7.1)	1.0 (0.3-3.2)

Odds ratios (ORs) and confidence interval (CI95%) for selected outcome measurements. The risk estimates are adjusted for age, sex, tumor and PTBE volume categories. Italics indicate statistically significant results. *Data not available for all patients

## Discussion

This study’s main findings were: 1) the mFI-5 and mFI-11 indices associate with postoperative mortality and surgery-associated complications in surgically treated supratentorial meningioma patients aged 80 years and older, and 2) even though frail and severely frail patients suffered from increased risk of experiencing surgery-associated complications, these patients also had the highest chance for major functional improvements.

In more detail, nearly one-third (mFI-5: 23.1%; mFI-11: 31.6%) of severely frail patients showed major functional improvements after surgery, and a quarter (mFI-5: 23.1%; mFI-11: 26.3%) gained functional independence (Table 3). On the other hand, a significant proportion of these patients (mFI-5: 42.9%; mFI-11: 31.0%) died within the first postoperative year (Table 3). Whether these results are acceptable or not, is debatable but it may be speculated that many severely frail patients would have died within a year even without surgery. Unfortunately, no natural history data of large or symptomatic meningiomas in this age group is available.

Optimal neurosurgical patient selection remains one of the key aspects of neuro-oncology, and is especially difficult in very old and often frail patients. Selection of the correct patients is essential in order to achieve good surgical outcome. In our opinion, the primary goal of surgery in these patients with an inherently limited life expectancy should not necessarily be prolonged overall survival, but surgical success should rather be measured by functional improvement and maintaining independence, which are often associated with a high quality of life. Classifications and indices, such as the mFI-5 and mFI-11, aim at facilitating and objectifying patient selection and individual risk/benefit analyses. The scores should be applied in the context of the surgeon’s own clinical experience and the patient’s individual situation. We consider these scores and predictions as very useful tools for making shared decisions with patients. In other words, calculating the scores could provide patients with more information as to what to expect from surgery, and hereby provide a better basis for informed decision-making.

Besides a scoring system’s correct risk assessment with regard to patient outcome, the applicability and ease of use are probably the main aspects when it comes to decide whether a scoring system can be implemented into clinical use. From a neurosurgeon’s point of view, the decision on whether or who to operate often have to be made within the timeframe of a single or few outpatient visits. Both, the mFI-5 and mFI-11 are based on the patients’ comorbidities and past medical history as well as functional status, which can be easily extracted from medical data, obtained by taking the patients’ medical history, and clinical assessment in an outpatient setting (Table 1). Therefore, both scores are potentially useful in the above laid out environment.

As stated above, patient frailty as assessed by the mFI-5 and mFI-11 associated well with mortality within the first postoperative year and occurrence for complications. Importantly, we were also able to determine that frail and severely frail patients, despite suffering from a relevant number of comorbidities, had a good chance of experiencing functional improvements and gaining functional independence after surgery. This is in line with our prior analysis, where also patients with poor functional status and large PTBE showed significant benefits from tumor removal [35, 36]. Thus, frailty should not deter per se from considering surgery in these very old patients. Nevertheless, careful consideration must be made in light of the increased risk for complications, and patients must be at the helm in deciding whether the potential functional benefit outweighs these risks. Furthermore, we found no significant differences in our association analyses between the mFI-5 and mFI-11. Thus, based on our assessment, there appears to be no clear additional benefit in using the more detailed mFI-11 over the mFI-5. In the context of existing literature, three other groups have previously evaluated the mFI-5 and mFI-11 in meningioma patients [21, 22, 34]. Ikawa et al. reported a mFI-5≥2 to be a significant risk factor for worsening Barthel Index, in-hospital mortality, and complications [21]. Contrary to our findings, however, they found the mFI-5 to be useful in younger patients rather than in elderly patients aged ≥75 years [21]. Cole et al. found that severely frail patients (i.e. mFI-5≥3) were at the highest risk for major complications, unplanned readmission or reoperation, and discharge other than home [22]. Dicpinigaitis et al. also reported that increasing frailty, assessed by the mFI-11, associated with complications and mortality [34]. While all these studies confirmed frailty to lead to increased complication and mortality rates, none specifically addressed the potential functional benefit from surgery in very old patients with high frailty scores. Besides microsurgery, stereotactic radiosurgery has been shown to be a viable alternative treatment option for meningiomas, which results in excellent tumor control and should therefore be considered in patients with significant frailty [38, 39]. Tumor volume, however, is often a limiting factor for radiosurgery. In our study, which focuses exclusively on surgical results, the majority of patients (92.5%) suffered from significant mass effect with neurological deficits, and a significant proportion of tumors (35.5%) had a diameter of ≥5cm rendering radiosurgery a suboptimal treatment option.

In the context of our previously introduced decision support tool, which was based on the patients’ preoperative functional status and PTBE volume, a somewhat similar pattern could be observed by the current frailty index analyses [35, 36]. Again, the subgroup of perceived “high-risk” patients with poor preoperative status, i.e. severely frail, patients showed the highest chance for postoperative functional improvements. However, whereas PTBE volume did not associate with an increased risk for surgery-associated complications, patient frailty was found to be a significant factor for increased risk of postoperative complications.

All things considered, we believe that the assessed frailty indices provide useful information when it comes to shared decision-making and counseling very old meningioma patients. Both indices are rather easy to apply and therefore time-efficient tools in neurosurgical oncology.

### Limitations

Data was collected retrospectively leading to different degrees of missing values for specific analyzed parameters and follow-up was not performed uniformly; detailed follow-up and outcome data has been previously published [36]. Indication for surgery may have differed between centers and no conclusions for conservatively treated patients can be drawn from our analysis. Additionally, it is possible that not all comorbidities were correctly recorded, which may lead to lower mFI scores and hereby cause an underestimation of the effect size. No standardized outcome assessment protocol with regard to recorded complications was in use in any of the participating study centers. This aspect would be beneficial for future analyses, as the potential benefits and drawbacks of surgical treatment in this particular patient group should be weighed up particularly carefully. With regard to the assessment of the patients’ capability to live at home after tumor resection, detailed information on whether patients living at home required extensive assistance was limited. Furthermore, statistical analyses of skullbase meningioma patients were hampered by the low sample size. Lastly, the natural course of symptomatic meningiomas in very old patients remains unknown and since our database does not included conservatively treated patients, no conclusions on that matter can be drawn from our analyses.

## Conclusions

The mFI-5 and mFI-11 are easily applicable scoring systems that associate well with postoperative mortality and surgery-associated complications in very old patients with surgically treated supratentorial meningiomas. Even though patients with significant comorbidities have the highest risk for surgery-associated complications, they also have a fair chance for postoperative functional improvements. Although mFI-11 considers more parameters, there is no advantage of the application compared to mFI-5.

## Supplementary information

Below is the link to the electronic supplementary material.

Supplementary file1 (DOCX 15 KB)

Supplementary file2 (DOCX 14 KB)

## Data Availability

No datasets were generated or analysed during the current study

## Abbreviations

CI: Confidence interval
COPD: Chronic obstructive pulmonary disease
KPS: Karnofsky Performance Scale
mFI-5: Modified 5 Factor Frailty Index
mFI-11: Modified 11 Factor Frailty Index
MRI: Magnetic resonance imaging
OR: Odds ratio
PTBE: Peritumoral brain edema
