# Supplementary material for: Frailty indices predict mortality, complications and functional improvements in supratentorial meningioma patients over 80 years of age
Source: J Neurooncol. 2024 Sep 4;170(1):89–100. doi: 10.1007/s11060-024-04780-6 (PMC11447097; doi:10.1007/s11060-024-04780-6)
Supplement: Supplementary file 1 — Supplementary file1 (DOCX 15 KB) [file 11060_2024_4780_MOESM1_ESM.docx]

**Supplementary Table 1 – Functional outcome, mortality, and complications for skullbase meningioma patients**

|  | **All patients (n=64)** | **Modified 5 Factor Frailty Index (mFI-5)** | | | | **Modified 11 Factor Frailty Index (mFI-11)** | | | |
| --- | --- | --- | --- | --- | --- | --- | --- | --- | --- |
|  |  | ***Robust “mFI=0” (n=8)*** | ***Pre-frail “mFI=1” (n=35)*** | ***Frail “mFI=2”***  ***(n=18)*** | ***Severely frail “mFI≥3” (n=3)*** | ***Robust “mFI=0” (n=6)*** | ***Pre-frail “mFI=1” (n=29)*** | ***Frail “mFI=2”***  ***(n=14)*** | ***Severely frail “mFI≥3” (n=15)*** |
| *Functional benefit at last follow-up within the first postoperative year* | | | | | | | | | |
| Any functional  improvement  (KPS≥10) (n): | 25 (39.1%) | 1 (12.5%) | 16 (45.7%) | 7 (38.9%) | 1 (33.3%) | 1 (16.5%) | 12 (41.4%) | 5 (35.7%) | 7 (46.7%) |
| Major  improvement  (KPS≥20) (n): | 15 (23.4%) | 1 (12.5%) | 8 (22.9%) | 5 (27.8%) | 1 (33.3%) | 1 (16.5%) | 7 (24.1%) | 2 (14.3%) | 5 (33.3%) |
| Unchanged  (n): | 22 (34.4%) | 7 (87.5%) | 11 (31.4%) | 4 (22.2%) | 0 (0.0%) | 5 (83.3%) | 10 (34.5%) | 2 (14.3%) | 5 (33.3%) |
| Worse (n): | 17 (26.6%) | 0 (0.0%) | 8 (22.9%) | 7 (38.9%) | 2 (66.7%) | 0 (0.0%) | 7 (24.1%) | 7 (50.0%) | 3 (20.0%) |
| *Functional independence (KPS≥70) at last follow-up within the first postoperative year* | | | | | | | | | |
| Gained (n): | 14 (21.9%) | 0 (0.0%) | 8 (22.9%) | 5 (27.8%) | 1 (33.3%) | 0 (0.0%) | 7 (24.1%) | 2 (14.3%) | 5 (33.3%) |
| Maintained  (n): | 30 (46.9%) | 8 (100.0%) | 19 (54.3%) | 2 (11.1%) | 1 (33.3%) | 6 (100.0%) | 15 (51.7%) | 5 (35.7%) | 4 (26.7%) |
| Neither (n): | 12 (18.8%) | 0 (0.0%) | 3 (8.6%) | 8 (44.4%) | 1 (33.3%) | 0 (0.0%) | 3 (10.3%) | 4 (28.6%) | 5 (33.3%) |
| Lost (n): | 8 (12.5%) | 0 (0.0%) | 5 (14.3%) | 3 (16.7%) | 0 (0.0%) | 0 (0.0%) | 4 (13.8%) | 3 (21.4%) | 1 (6.7%) |
| *Postoperative mortality, surgery-associated complications and postoperative new deficits** | | | | | | | | | |
| Within 90-  days (n): | 3 (4.7%) | 1 (12.5%) | 1 (3.3%) | 1 (5.9%) | 1 (33.3%) | 0 (0.0%) | 1 (4.3%) | 1 (7.1%) | 1 (7.1%) |
| Within 12-  months (n): | 3 (4.7%) | 2 (25.0%) | 1 (3.8%) | 1 (8.3%) | 1 (50.0%) | 0 (0.0%) | 1 (5.0%) | 1 (8.3%) | 1 (12.5%) |
| Complications  (n): | 30 (46.9%) | 1 (12.5%) | 18 (51.4%) | 9 (50.0%) | 2 (66.7%) | 1 (16.7%) | 14 (48.3%) | 8 (57.1%) | 7 (46.7%) |
| New deficits  (n): | 11 (17.2%) | 0 (0.0%) | 7 (20.0%= | 2 (11.1%) | 2 (66.7%) | 0 (0.0%) | 6 (20.7%) | 2 (14.3%) | 3 (20.0%) |
| *Patients’ ability to live at home after surgery** | | | | | | | | | |
| Living at home  (n): | 30 (46.9%) | 4 (50.0%) | 21 (95.5%) | 5 (83.3%) | 0 (0.0%) | 4 (100.0%) | 14 (100.0%) | 7 (87.5%) | 5 (62.5%) |

**Data not available for all patient subgroups; percentages calculated for available valid data*
