# Supplementary material for: Frailty indices predict mortality, complications and functional improvements in supratentorial meningioma patients over 80 years of age
Source: J Neurooncol. 2024 Sep 4;170(1):89–100. doi: 10.1007/s11060-024-04780-6 (PMC11447097; doi:10.1007/s11060-024-04780-6)
Supplement: Supplementary file 2 — Supplementary file2 (DOCX 14 KB) [file 11060_2024_4780_MOESM2_ESM.docx]

**Supplementary Table 2 – Statistics for skullbase meningioma patients**

| ***Modified 5 Factor Frailty Index (mFI-5)*** | | | | | | | | |  |  |  |  |
| --- | --- | --- | --- | --- | --- | --- | --- | --- | --- | --- | --- | --- |
| **Parameter** | **Any functional**  **Improvement**  **(KPS≥10)** | **Major**  **improvement (KPS≥20)** | **Gained/maintained functional independence (KPS≥70)** | **90-day mortality*** | **1-year mortality*** | **Surgery-associated complications** | **New neurological deficits** | **Capability to live at home*** |  |  |  |  |
| Robust “mFI=0”: | [Reference]  OR (CI95%) | [Reference]  OR (CI95%) | [Reference]  OR (CI95%) | [Reference]  OR (CI95%) | [Reference]  OR (CI95%) | [Reference]  OR (CI95%) | [Reference]  OR (CI95%) | [Reference]  OR (CI95%) |  |  |  |  |
| Pre-frail “mFI=1”: | 6.8 (0.6-78.5) | 2.6 (0.2-34.3) | 0.0 (0.0-not reached) | 70797938.8 (0.0-not reached) | 80539210.9 (0.0-not reached) | 11.8 (1.1-129.4) | 958339349.7 (0.0-not reached) | 0.0 (0.0-not reached) |  |  |  |  |
| Frail “mFI=2”: | 4.0 (0.3-48.0) | 2.5 (0.2-33.5) | 0.0 (0.0-not reached) | 20036759.4 | 48064349.8 (0.0-not reached) | 8.6 (0.7-103.4) | 333954363.6 (0.0-not reached) | 0.0 (0.0-not reached) |  |  |  |  |
| Severely frail “mFI≥3”: | 4.0 (0.1-116.1) | 4.5 (0.1-160.9) | 0.0 (0.0-not reached) | 821284808.9 (0.0-not reached) | 852804187.8 (0.0-not reached) | 23.0 (0.8-675.0) | 11682386092.8 (0.0-not reached) | 0.0 (0.0-not reached) |  |  |  |  |
| ***Modified 11 Factor Frailty Index (mFI-11)*** | | | | | | | | |  | 1,147 | ,788 | 1,670 |
| Robust “mFI=0”: | [Reference]  OR (CI95%) | [Reference]  OR (CI95%) | [Reference]  OR (CI95%) | [Reference]  OR (CI95%) | [Reference]  OR (CI95%) | [Reference]  OR (CI95%) | [Reference]  OR (CI95%) | [Reference]  OR (CI95%) |  |  |  |  |
| Pre-frail “mFI=1”: | 3.5 (0.3-43.4) | 1.7 (0.1-26.2) | 0.0 (0.0-not reached) | 147517471.5 (0.0-not reached) | 155857091.1 (0.0-not reached) | 7.4 (7.4-91.2) | 888107491.4 (0.0-not reached) | 19512.8 (0.0-not reached) |  |  |  |  |
| Frail “mFI=2”: | 1.2 (0.1-17.4) | 0.5 (0.0-9.6) | 0.0 (0.0-not reached) | 31456995.0 (0.0-not reached) | 20757895.3 (0.0-not reached) | 9.7 (9.7-142.8) | 321150850.7 (0.0-not reached) | 0.0 (0.0-not reached) |  |  |  |  |
| Severely frail “mFI≥3”: | 4.6 (0.3-66.9) | 4.1 (0.2-76.7) | 0.0 (0.0-not reached) | 133931086.7 (0.0-not reached) | 380460017.8 (0.0-not reached) | 6.5 (6.5-91.8) | 951476944.5 (0.0-not reached) | 0.0 (0.0-not reached) |  |  |  |  |

Odds ratios (ORs) and confidence interval (CI95%) for selected outcome measurements. The risk estimates are adjusted for age, sex, tumor and PTBE volume categories. Italics indicate statistically significant results. *Data not available for all patients.
